# Supplementary material for: Non-Ventilated Patients with Spontaneous Pneumothorax or Pneumomediastinum Associated with COVID-19: Three-Year Debriefing across Five Pandemic Waves
Source: J Pers Med. 2023 Oct 15;13(10):1497. doi: 10.3390/jpm13101497 (PMC10608223; doi:10.3390/jpm13101497)
Supplement: Supplementary file 1 [file jpm-13-01497-s001.zip › jpm-2643793-supplementary.pdf]

Supplementary Table S1. The most frequent symptoms and comorbidities observed in SP–SPM patients.

Variable[(a)]		All waves (N=190)	Wave 1 (N = 15)	Wave 2 (N = 32)	Wave 3 (N = 46)	Wave 4 (N = 29)	Wave 5 (N = 68)	p-value[(b)]
**Symptoms**								
Cough	+COVID-19	26 (40%)	–	11 (42.3%)	10 (38.5%)	2 (7.7%)	3 (11.5%)	0.007**
	-COVID-19	39 (60%)	7 (17.9%)	5 (12.8%)	10 (25.6%)	4 (10.3%)	13 (33.3%)	
Dyspnea	+COVID-19	40 (26.6%)	–	17 (42.5%)	14 (35%)	2 (5%)	7 (17.5%)	<0.001**
	-COVID-19	110 (73.4%)	15 (13.6%)	12 (10.9%)	24 (21.8%)	15 (13.6%)	44 (40%)	
Chest pain	+COVID-19	36 (30.3%)	–	15 (41.7%)	13 (36.1%)	2 (5.6%)	6 (16.7%)	<0.001**
	-COVID-19	83 (69.7%)	10 (12%)	10 (12%)	22 (26.5%)	11 (13.3%)	30 (36.1%)	
Tiredness	+COVID-19	28 (48.3%)	–	12 (42.9%)	10 (35.7%)	1 (3.6%)	5 (17.9%)	0.011*
	-COVID-19	30 (51.7%)	4 (13.3%)	6 (20%)	4 (13.3%)	3 (10%)	13 (42.3%)	
**Comorbidities**								
COPD	+COVID-19	6 (24%)	–	2 (33.3%)	1 (16.7%)	-	3 (50%)	0.307
	-COVID-19	19 (76%)	5 (26.3%)	2 (10.5%)	1 (5.3%)	3 (15.8%)	8 (42.1%)	
Asthma	+COVID-19	7 (66.7%)	–	2 (28.6%)	3 (42.9%)	-	2 (28.6%)	0.757
	-COVID-19	4 (33.3%)	–	1 (25%)	1 (25%)	-	2 (50%)	
Hypertension	+COVID-19	26 (46.4%)	–	11 (42.3%)	10 (38.5%)	-	5 (19.2%)	0.011*
	-COVID-19	30 (53.6%)	3 (10%)	4 (13.3%)	7 (23.3%)	3 (10%)	13 (43.3%)	
DM	+COVID-19	8 (50%)	–	2 (25%)	2 (25%)	1 (12.5%)	3 (37.5%)	1
	-COVID-19	8 (50%)	–	2 (25%)	2 (25%)	1 (12.5%)	3 (37.5%)	
Obesity	+COVID-19	3 (33.3%)	–	-	2 (66.7%)	1 (33.3%)	-	0.392
	-COVID-19	6 (66.7%)	–	1 (16.7%)	1 (16.7%)	2 (33.3%)	2 (33.3%)	
CKD	+COVID-19	6 (66.7%)	–	2 (33.3%)	3 (50%)	1 (16.7%)	-	0.741
	-COVID-19	3 (33.3%)	–	1 (33.3%)	2 (66.7%)	-	-	
Active cancer	+COVID-19	3 (16.7%)	–	2 (66.7%)	-	-	1 (33.3%)	0.355
	-COVID-19	15 (83.3%)	1 (6.7%)	2 (13.3%)	2 (13.3%)	2 (13.3%)	8 (53.3%)	

[(a)] Observed frequency (percent). Percentages:"All patients" column shows percentages based on the total number of patients presenting each symptom or comorbidity, all other columns show percentages based on the row total.

[(b)] Chi-square test (variables' distribution between COVID-19 and non-COVID-19 cases across pandemic waves).

*,** Statistical significance, $p < 0.05$, $p < 0.01$

Supplementary Table S2. Localization and extension of pneumothorax.

Variable[(a)]		All waves (N = 190)	Wave 1 (N = 15)	Wave 2 (N = 32)	Wave 3 (N = 46)	Wave 4 (N = 29)	Wave 5 (N = 68)	p-value[(b)]
Right pneumothorax	+COVID-19	28 (25.5%)	–	11 (55%)	7 (24.1%)	2 (12.5%)	8 (19.5%)	0.488
	-COVID-19	82 (74.5%)	4 (100%)	9 (45%)	22 (75.9%)	14 (87.5%)	33 (80.5%)	
Left pneumothorax	+COVID-19	12 (18.2%)	–	5 (50%)	3 (25%)	1 (11.1%)	3 (13%)	0.038*
	-COVID-19	54 (81.8%)	12 (100%)	5 (50%)	9 (75%)	8 (88.9%)	20 (87%)	
**Extension of pneumothorax**								
no pneumothorax	+COVID-19	12 (54.5%)	–	3 (100%)	6 (85.7%)	1 (50%)	2 (20%)	0.004**
	-COVID-19	10 (45.5%)	–	-	1 (14.3%)	1 (50%)	8 (80%)	
< 10%	+COVID-19	12 (35.3%)	–	6 (75%)	1 (14.3%)	2 (40%)	3 (25%)	
	-COVID-19	22 (64.7%)	2 (100%)	2 (25%)	6 (85.7%)	3 (60%)	9 (75%)	
10-50%	+COVID-19	17 (18.1%)	–	3 (30%)	5 (26.3%)	2 (11.1%)	7 (17.1%)	
	-COVID-19	77 (81.9%)	6 (100%)	7 (70%)	14 (73.7%)	16 (88.9%)	34 (82.9%)	
> 50%	+COVID-19	11 (27.5%)	–	6 (54.5%)	3 (23.1%)	1 (25%)	1 (20%)	
	-COVID-19	29 (72.5%)	7 (100%)	5 (45.5%)	10 (76.9%)	3 (75%)	4 (80%)	

[(a)] Observed frequency (percent). Percentages:"All patients" column shows percentages based on the total number of patients presenting each symptom or comorbidity, all other columns show percentages based on the row total.

[(b)] Chi-square test (variables' distribution between COVID-19 and non-COVID-19 cases across pandemic waves).

*,** Statistical significance, $p < 0.05$, $p < 0.01$

Supplementary Table S3. Laboratory findings for COVID-19 patients and non-COVID-19 patients across pandemic waves.

Variable[(a)]		All waves (N = 190)	Wave 1 (N = 14)	Wave 2 (N = 34)	Wave 3 (N = 45)	Wave 4 (N = 29)	Wave 5 (N = 68)	p-value[(b)]
CRP [mg/L]	+COVID-19	109.5 (63 – 172.9)	–	82.5 (31 – 146)	109 (78.6 – 146.79)	172.9 (70.1 – 202.5)	155.05 (91.6 – 187.65)	0.42
	-COVID-19	55.9 (6.7 – 101.5)	–	10 (8.65 – 55.65)	51 (1 – 192)	5.1 (1.1 – 85.4)	18.34 (6.1 – 81.5)	0.973
CRT [mg/dl]	+COVID-19	1.01 (0.85 – 1.34)	–	0.98 (0.76 – 1.04)	1.05 (0.92 – 1.36)	1.33 (1.2 – 2.3)	1.04 (0.9 - 1.14)	0.248
	-COVID-19	0.81 (0.67 – 1.11)	0.89 (0.73 – 1.07)	0.8 (0.62 – 1)	0.92 (0.8 – 1.13)	0.84 (0.67 – 0.94)	0.9 (0.75 – 1.16)	0.279
Urea [mg/dl]	+COVID-19	49 (34 - 71)	–	39 (30 - 52)	62 (44.5 – 72.5)	85 (74 - 186)	42 (31 – 64.85)	0.033*
	-COVID-19	35.8 (24 – 55.95)	32 (27.5 – 37.5)	29.5 (20 – 46)	32 (25 – 42)	31 (29 – 43)	34 (26 – 56)	0.868
WBC [x10³ /μL]	+COVID-19	9.75 (6.1 – 15.7)	–	9.6 (7.1 – 14.3)	7.9 (6.57 – 16.65)	6.1 (6 – 9.9)	14.15 (8.6 – 15.8)	0.334
	-COVID-19	8.61 (7.39 – 11.9)	8.9 (8.1 – 12.3)	10.38 (7.8 – 17.3)	9.7 (8.6 – 12.6)	7.8 (6.8 – 10.7)	11.7 (8.9 – 13.67)	0.280
Lymph [x10³ /μl]	+COVID-19	1 (0.7 – 1.3)	–	1.07 (0.7 – 1.67)	1 (0.68 – 1.2)	0.8 (0.6 - 1)	0.92 (0.75 – 1.35)	0.4
	-COVID-19	1.7 (1.25 – 2.1)	1.9 (1.45 – 2.1)	1.87 (1.39 – 2.1)	2 (1.63 – 2.41)	1.91 (1.7 – 2.54)	1.8 (1.25 – 3.1)	0.847
PLT [x10³ /μL]	+COVID-19	242.5 (178 - 354)	–	242.5 (215 - 358)	172 (146.5 - 289)	238 (216 - 285)	388 (311 - 411)	0.038*
	-COVID-19	281 (236.5 – 356)	314 (238 – 389.5)	300 (245 – 360)	261 (228 - 334)	243 (158 – 280)	264 (212 – 302)	0.161
D-dimer [ng/ml]	+COVID-19	1582.2 (932 - 2852)	–	2284 (932 - 3620)	1480 (998.5 – 2872.5)	1250 (810 - 1570)	1965 (955 - 5780)	0.656
	-COVID-19	2200 (830 - 3440)	–	1846 (552 – 6882)	1540 (525 – 6882)	2020 (1217 – 3730)	2220 (690 – 3260)	0.753
GLI [mg/dl]	+COVID-19	126 (105 -177)	–	126 (103 - 139)	124 (107.5 - 154)	186 (158 - 313)	133.5 (113 - 175)	0.446
	-COVID-19	126 (97.5 – 134)	111 (95.5 – 126)	123 (99 – 146)	103 (94 – 131.5)	100.5 (97 – 118)	113 (99 – 133)	0.366

[(b)]median (interquartile range),

[(b)]Kruskal-Wallis nonparametric test (separate for each row).

* Statistical significance, $p < 0.05$.
